# Whole-genome sequencing analysis of *Salmonella enterica* serotype Enteritidis isolated from poultry sources in Mongolia

**DOI:** 10.3389/fvets.2025.1595674

**Published:** 2025-05-20

**Authors:** Seung-un Song, Tae-Min La, Taesoo Kim, Junyoung Kim, Eunkyung Shin, Uyangaa Temuujin, Ji-Yeon Hyeon, Dong-Hun Lee, Sang-Won Lee

**Affiliations:** ^1^College of Veterinary Medicine, Konkuk University, Seoul, Republic of Korea; ^2^Division of Bacterial Diseases, Bureau of Infectious Disease Diagnosis Control, Korea Disease Control and Prevention Agency, Cheongju, Republic of Korea; ^3^School of Veterinary Medicine, Mongolian University of Life Sciences, Ulaanbaatar, Mongolia

**Keywords:** *Salmonella* Enteritidis, antimicrobial susceptibility test, whole-genome sequencing, antimicrobial resistance gene, whole-genome SNP analysis, poultry

## Abstract

*Salmonella enterica* serotype Enteritidis (*S.* Enteritidis) is a leading foodborne pathogen associated with poultry products, and the emergence of antimicrobial resistance (AMR) in this serotype poses a growing public health concern, particularly in regions with increasing poultry trade. Between April and June 2024, we collected 114 poultry meat samples (Mongolian domestic and Chinese imported) from retail markets in Mongolia and isolated 45 *S.* Enteritidis strains. Antimicrobial susceptibility testing revealed high resistance rates to nalidixic acid (100%), ampicillin (93.3%), and streptomycin (88.9%). Whole-genome sequencing (WGS) identified major resistance genes, including *aac(6′)-Iaa*, *aph(3″)-Ib*, *bla*TEM-1B, and *sul2*. Mongolian domestic isolates additionally harbored extended-spectrum *β*-lactamase (ESBL) *bla*CTX-M-14 and plasmid-mediated quinolone resistance (PMQR) *qnrS1,* both of which are clinically significant. Plasmid replicon typing revealed IncF as the most prevalent type across isolates, while IncI1- *α* was predominantly found in multidrug-resistant (MDR) domestic strains. Phylogenetic analysis using whole-genome SNPs (wgSNPs) demonstrated that domestic and imported isolates clustered separately, indicating that Chinese *S.* Enteritidis strains have not yet been introduced into Mongolia’s domestic poultry industry. This study represents the first comprehensive analysis of prevalence and resistance mechanisms of *S.* Enteritidis in Mongolia poultry production. The findings underscore the necessity of continuous surveillance and the implementation of effective antibiotic stewardship in the poultry production sector.

## 1 Introduction

Non-typhoidal *Salmonella enterica* (*S. enterica*) is a major foodborne pathogen that causes salmonellosis worldwide (1). Among its serotypes, *Salmonella* Enteritidis (*S.* Enteritidis) is a leading serovar and is a major cause of foodborne illness, particularly primarily transmitted through poultry products such as chicken meat and eggs (2, 3). According to the Centers for Disease Control and Prevention (CDC), *S.* Enteritidis infections have increased globally, particularly in regions where poultry consumption is widespread (4).

Mongolia has traditionally relied on locally sourced meats such as lamb, goat, and beef, supported by its robust pastoral economy. According to the Mongolian Meat Association, the country produced 420,000 tons of meat in 2017, with per capita meat consumption reaching 16.4 kg, reflecting increased demand driven by economic growth and rising living standards (5). Recently, changing dietary habits and urbanization have led to growing poultry consumption in Mongolia, despite limited local poultry production capacity. Poultry meat, however, constitutes only a minor fraction of domestic production, highlighting Mongolia’s heavy reliance on imports. FAOSTAT data indicate that Mongolia’s chicken meat imports from China have steadily increased, rising more than five-fold from 2015 to 2022. This dependence on Chinese poultry raises food safety concerns, as studies have identified a growing prevalence of multidrug-resistant (MDR) *Salmonella* in Chinese poultry production (6–8). Such reliance increases the risk of introducing antimicrobial-resistant *S.* Enteritidis into Mongolia’s food supply, posing substantial threats to public health.

Recent advances in whole-genome sequencing (WGS) technology and bioinformatics have provided powerful tools for genetic analysis of *Salmonella,* studying public health surveillance, antimicrobial resistance detection, and epidemiological tracking (9–11). However, no WGS-based studies on *S.* Enteritidis have been conducted in Mongolia, leaving its prevalence and antimicrobial resistance profiles in the local food supply chain unexplored. A previous study in Mongolia employed direct polymerase chain reaction (PCR) to assess microbial contamination in poultry meat, reporting a *Salmonella* spp. prevalence of 7.6% (12). Given Mongolia’s heavy reliance on poultry imports from China, there is a critical need to investigate whether MDR *S.* Enteritidis strains are being introduced and established in the national poultry industry. Therefore, this study aims to employ WGS to investigate the prevalence and resistance profiles of *Salmonella* spp., representing the first genomic assessment of MDR *S.* Enteritidis in poultry meat sold in Mongolian retail markets.

In this study, we collected poultry meat samples from Mongolian retail markets, including both domestic products and imports from China and Russia, to determine the prevalence of *Salmonella* spp. in the national poultry supply. Additionally, we employed WGS to characterize antimicrobial resistance profiles, plasmid types, and phylogenetic relationships among *S.* Enteritidis isolates. This research represents the first WGS-based prevalence study of *S.* Enteritidis in Mongolia’s poultry supply chain.

## 2 Materials and methods

### 2.1 *Salmonella* isolation from poultry sources

From April 15th to June 17th, 2024, a total of 114 samples of retail chicken meats were collected and tested, all purchased from retail markets in Ulaanbaatar, Mongolia. These included domestic Mongolian whole chickens and imported Chinese and Russian sliced chicken meats. Retail chicken meats were aseptically placed in a sterile plastic bag containing 400 mL of buffered peptone water broth (BPW, Difco, Detroit, MI, USA) and shaken for 2 mins. From this initial rinse, a 20 mL aliquot of the BPW-sample mixture was transferred, vortex-mixed with fresh 20 mL of BPW for 15 s, then incubated at 36 ± 1°C for 18–24 h. Subsequently, 100 μL of the incubated culture was vortex-mixed for 15 s in 10 mL of Rappaport-Vassiliadis (RV) broth, then incubated at 41.5 ± 1°C for 20–24 h. Cultures exhibiting a color change were streaked onto *Salmonella* ChromoSelect agar (Sigma-Aldrich, St. Louis, MO, USA) and incubated at 37°C for 24 h. Pink colonies presumptively identified as *Salmonella* spp. on the agar were confirmed using the VITEK-MS system (bioMérieux, Marcy-L’Etoile, France). Confirmed isolates were stored at −80°C in glycerol. All *Salmonella* spp. isolates obtained during the study period were included for the further studies, following their transport to Konkuk University, Seoul, Korea.

### 2.2 Whole-genome sequencing and genome assembly

Genomic DNA was extracted from overnight cultures of *Salmonella* spp. using the QIAamp DNA Mini Kit (Qiagen, Hilden, Germany) following the manufacturer’s instructions. The purity and concentration of the extracted DNA were measured with the NanoDrop™ spectrophotometer (Thermo Fisher Scientific) and the Quantus™ fluorometer (Promega, Madison, WI, USA), respectively. Library preparation was performed with the TruSeq Nano DNA Library Prep kit (Illumina, San Diego, CA, USA) following the manufacturer’s instructions. The library was then sequenced on a NovaSeq 6,000 platform (Illumina, San Diego, CA, USA), generating 150 bp paired-end reads. Read quality was assessed using fastp v.0.20.1 (13). Low-quality bases and adapter sequences were trimmed using Trimmomatic v0.39 (14). Trimmed reads were assembled using SPAdes v4.1.0 (15) with the “—careful” option. Serotype prediction was performed using SeqSero v1.3.1 (16) on the assembled contigs, and only isolates identified as serovar Enteritidis were included in further analyses. The whole-genome sequencing data generated in this study has been deposited in the NCBI BioProject database under accession number PRJNA1236736.

### 2.3 Antimicrobial susceptibility test

Antimicrobial susceptibility testing was determined using the Sensititre™ ARIS HiQ AST system (Thermo Fisher Scientific, Waltham, MA, USA) with the Sensititre™ panel (KRCDC2F; Thermo Fisher Scientific, Waltham, MA, USA). The following antimicrobials were tested: ciprofloxacin (CIP, 0.03–0.5 μg), nalidixic acid (NAL, 2–128 μg), imipenem (IMI, 1–8 μg), colistin (COL, 2–16 μg), ampicillin (AMP, 2–64 μg), tetracycline (TET, 2–128 μg), chloramphenicol (CHL, 2–32 μg), azithromycin (AZI, 2–32 μg), gentamicin (GEN, 1–64 μg), streptomycin (STR, 2–128 μg; Ref. National Antimicrobial Resistance Monitoring System, 2014) (17), amikacin (AMI, 4–64 μg), trimethoprim/sulfamethoxazole (SXT, 1/19–16/304), cefotaxime (FOT, 1–32 μg), ceftriaxone (AXO, 1–32 μg), cefoxitin (FOX, 4–32 μg), and ceftazidime (TAZ, 1–16 μg).

Purified bacterial isolates were suspended in distilled pure water and adjusted to a 0.5 McFarland standard using a nephelometer. A 10 μL aliquot of the adjusted suspension was inoculated into 11 mL of cation-adjusted Mueller-Hinton (MH) medium and dispensed into the KRCDC2F Sensititre™ panel using the Sensititre AIM automated dispensing system. The inoculated microplates were sealed with a microplate-sealing film and incubated at 37°C for 18 h in the Sensititre ARIS HiQ automated analysis system.

After incubation, a fluorescence auto-read system determined minimum inhibitory concentration (MIC) values. These MIC values were determined according to Clinical and Laboratory Standards Institute (CLSI) M100-2018 28th edition guidelines (Wayne, PA, USA) (18), except for Streptomycin, which followed the National Antimicrobial Resistance Monitoring System (NARMS) criteria (17). *Escherichia coli* ATCC25922 was used as a quality control standard. *Salmonella* isolates resistant to more than three classes and more than one antimicrobial in a single class were designated MDR strains, following standard criteria (19).

### 2.4 Phylogenetic analysis

Whole-genome single nucleotide polymorphisms (wgSNPs) were identified among *S.* Enteritidis (*n* = 45) in this study using Snippy v4.6.0^1^ (20), with *S.* Enteritidis strain P125109 as the reference for SNP calling (GenBank accession no. AM933172.1, NCBI RefSeq accession no.NC_011294.1). Prophage regions, repetitive genomic regions, and recombination regions in the reference genome were identified and excluded from the analysis. Specifically, prophage regions were identified using PHASTEST (21), repetitive genomic regions were determined by self-alignment of the reference genome using the nucmer tool from the Mummer package v.4.0.1 (22) using the --maxmatch and --nosimplify options, and recombination regions were filtered out using Gubbins v.3.4 (23). A maximum likelihood phylogenetic tree was constructed from the cleaned alignment using a TVMe model selected by ModelFinder (24) in IQ-TREE (25) under 1,000 bootstrap replicates. The phylogenetic tree was visualized using the interactive Tree of Life version 5 (iTOLv7)^2^. Genotype clustering was performed using Bayesian Analysis of Population Structure (BAPS) through the “FastBAPS” R package.

For phylogenetic analysis with Chinese isolates, genomic sequences of *S.* Enteritidis isolates (*n* = 816) from China were downloaded from EnteroBase^3^ (26). A phylogenetic tree was constructed using Mashtree v.1.4.6 (27) with bootstrapping using Mash distances. To reduce redundancy and computational complexity, Treemmer v0.3 (28) was applied with the -lmc option, pruning the tree to maintain its overall topology while preserving all Mongolian *S.* Enteritidis isolates and selectively reducing the number of Chinese isolates by removing leaves that contribute minimally to the tree’s diversity.

The final dataset included a total of 106 isolates comprising 45 Mongolian isolates from this study and 61 Chinese isolates (detailed in Supplementary Table S3). These isolates were analyzed using wgSNP, followed the construction of a maximum-likelihood phylogenetic tree using a TVM + F + ASC model, selected by ModelFinder (24) in IQ-TREE (25) under 1,000 bootstrap replicates and determination of population structure with FastBAPS as detailed above (29).

### 2.5 Antimicrobial resistance genes and comparative genome analysis

All isolates were screened for antimicrobial resistance genes (ARGs) using a pipeline available at github.com/maxlcummins/pipelord. Gene screening was performed using abricate v1.0.1^4^ in conjunction with the following databases: ResFinder (30) and PlasmidFinder (31). Genes were marked as present when detected by abricate and filtered using a custom R script^5^ to have 90% identity and 90% coverage.

## 3 Results

### 3.1 Prevalence of *Salmonella* spp.

A total of 56 *Salmonella* spp. isolates were recovered from 114 retail chicken meat samples: 48 isolates (63.2%, 48/76) from Mongolian domestic whole chickens and eight isolates (23.5%, 8/34) from Chinese imported sliced chicken meats. No *Salmonella* spp. was detected in Russian imported sliced chicken meat (*n* = 4). Sampling metadata for all *Salmonella* spp. are provided in Supplementary Table S1.

### 3.2 Serovars prediction

Whole-genome sequencing (WGS) analysis identified four distinct serovars among the 56 studied *Salmonella* isolates (Table 1). The predominant serovar was *S.* Enteritidis (80.4% 45/56), with 41 isolates recovered from Mongolian domestic chicken and four from Chinese imported chicken. *S.* Typhimurium (12.5% 7/56) was detected exclusively in Mongolian domestic chicken, while *S.* Agona (3.6% 2/56) and *S.* Indiana (3.6% 2/56) were only identified in imported samples from China.

**Table 1:** Prevalence and predicted serovar distribution of 56 *Salmonella* spp. isolates from Mongolian domestic and Chinese imported retail chicken meats collected in Ulaanbaatar, Mongolia, from April 15th to June 17th, 2024.

Predicted serovar^a^	Sample no. of Mongolian domestic chicken	Sample no. of Chinese imported chicken	Total no. of strain (%)
Enteritidis	41	4	45 (80.4%)
Typhimurium	7	0	7 (12.5%)
Agona	0	2	2 (3.6%)
Indiana	0	2	2 (3.6%)
Total	48	8	56 (100%)

^a^*Salmonella* spp. serovar prediction was performed using SeqSero.

### 3.3 Antimicrobial resistance profiles of *S.* Enteritidis

The antimicrobial resistance profiles of 45 *S.* Enteritidis isolates were determined using the broth dilution method against 16 antimicrobial agents, and the results are presented in Table 2 (Supplementary Table S2). The highest resistance was observed against nalidixic acid (100% 45/45), followed by ampicillin (93.3% 42/45), and streptomycin (88.9% 40/45). Resistance to third-generation cephalosporins (cefotaxime and ceftriaxone) was observed in 4 isolates (8.9%), all of which concurrently resistant to ampicillin, nalidixic acid, streptomycin, tetracycline, and trimethoprim/sulfamethoxazole (Table 2). Colistin resistance was also detected in four isolates (8.9%), two originating from Mongolia domestic chicken and the other two from imported Chinese chicken. The remaining 41 isolates (91.1%) were classified as intermediate to colistin.

**Table 2:** Antimicrobial resistance profiles of 45 *S.* Enteritidis isolates in this study tested against 16 antimicrobial agents using the Sensititre™ ARIS HiQ AST system.

Antimicrobial agent	Results of antimicrobial susceptibility in percentage (%)^a^		
	S	I	R
Penicillins			
Ampicillin (AMP)	3 (6.7%)	0 (0%)	42 (93.3%)
β-Lactams			
Cefotaxime (FOT)	41 (91.1%)	0 (0%)	4 (8.9%)
Cefoxitin (FOX)	45 (100%)	0 (0%)	0 (0%)
Ceftazidime (CAZ)	45 (100%)	0 (0%)	0 (0%)
Ceftriaxone (AXO)	41 (91.1%)	0 (0%)	4 (8.9%)
Carbapenems			
Imipenem (IMI)	45 (100%)	0 (0%)	0 (0%)
Aminoglycosides			
Gentamicin (GEN)	44 (97.8%)	0 (0%)	1 (2.2%)
Amikacin (AMI)	45 (100%)	0 (0%)	0 (0%)
Streptomycin (STR)^b^	5 (11.1%)	0 (0%)	40 (88.9%)
Macrolides			
Azithromycin (AZI)	45 (100%)	0 (0%)	0 (0%)
Tetracyclines			
Tetracycline (TET)	41 (91.1%)	0 (0%)	4 (8.9%)
Quinolones			
Ciprofloxacin (CIP)	44 (97.8%)	0 (0%)	1 (2.2%)
Nalidixic acid (NAL)	0 (0%)	0 (0%)	45 (100%)
Folate pathway inhibitor			
Trimethoprim/Sulfamethoxazole (SXT)	41 (91.1%)	0 (0%)	4 (8.9%)
Penicols			
Chloramphenicol (CHL)	45 (100%)	0 (0%)	0 (0%)
Polymyxin			
Colistin (COL)	0 (0%)	41 (91.1%)	4 (8.9%)

^a^ S, susceptible; I, intermediate; R, resistant.^b^ Streptomycin Ref. national antimicrobial resistance monitoring system (NARMS) 2014.

### 3.4 Antimicrobial resistance gene profile in *S.* Enteritidis

The WGS analysis results for the 45 *S.* Enteritidis isolates in this study are summarized in Table 3 (Supplementary Table S4). The most frequently detected resistance genes were *aac(6′)-Iaa* (100% 45/45), *bla*TEM-1B (93.3% 42/45), *aph(3″)-Ib* (88.9% 40/45), and *sul2* (88.9% 40/45), which confer resistance to aminoglycosides, penicillins, and sulfonamides, respectively. The *bla*CTX-M-14 gene, which confers resistance to third-generation cephalosporins, was detected in 4 isolates (8.9%). These isolates are harbored *aadA5, dfrA17, fosA3, qnrS1, and tet(A).*

**Table 3:** Antimicrobial resistance and MDR pattern in 45 *S.* Enteritidis isolate in this study.

Antimicrobial resistance profile	Antimicrobial resistance genes						Chromosome mutations	No. of isolates (%)	Co-resistant^a^	MDR status
	β-Lactam	Aminoglycoside	Fosfomycin	Tetracycline	Quinolone	Folate pathway inhibitor	Quinolone			
AMP-NAL-STR	*bla*TEM-1B	*aac(6′)-Iaa, aph(3″)-Ib, aph(6)-Id*				*sul2*	*gyrA* D87Y	34 (78.6%)	3	MDR
AMP-NAL-STR	*bla*TEM-1B	*aac(6′)-Iaa, aph(3″)-Ib*				*sul2*	*gyrA* D87Y, *gyrA* S83Y	1 (3.0%)	3	MDR
AMP-FOX-AXO-NAL-STR-TET-SXT	*bla*TEM-1B*bla*CTX-M-14	*aac(6′)-Iaa, aadA5,*	*fosA3*	*tet(A)*	*qnrS1*	*sul2, dfrA17*	*gyrA* D87Y	4 (8.9%)	5	MDR
		*aph(3″)-Ib, aph(6)-Id,*								
AMP-COL-NAL-STR	*bla*TEM-1B	*aac(6′)-Iaa, aph(3″)-Ib*				*sul2*	*gyrA* D87Y	1 (2.2%)	4	MDR
AMP-COL-NAL-STR	*bla*TEM-1B	*aac(6′)-Iaa*					*gyrA* D87Y	1 (2.2%)	4	MDR
AMP-GEN-NAL	*bla*TEM-1B	*aac(3)-IId,*					*gyrA* S83Y	1 (2.2%)	3	MDR
		*aac(6′)-Iaa,*								
		*aph(3′)-IIa*								
COL-NAL		*aac(6′)-Iaa*					*gyrA* D87Y	1 (2.2%)	2	Non-MDR
COL-NAL		*aac(6′)-Iaa*					*gyrA* D87N	1 (2.2%)	2	Non-MDR
NAL		*aac(6′)-Iaa*					*gyrA* D87N	1 (2.2%)	1	Non-MDR

Ampicillin (AMP), nalidixic acid (NAL), streptomycin (STR), cefoxitin (FOX), ceftriaxone (AXO), tetracycline (TET), trimethoprim/sulfamethoxazole (SXT), colistin (COL), gentamicin (GEN). ^a^Co-resistant refers to the number of antibiotic classes to which each isolate exhibits resistance, as determined by antimicrobial susceptibility testing.

In addition to antimicrobial resistance gene detection, chromosomal mutations associated with quinolone resistance were also investigated in Table 3. All isolates carried at least one non-synonymous point mutation in the *gyrA* gene including *gyrA* D87Y (Aspartic acid to Tyrosine) in 93.3% (42/45) of isolates, *gyrA* D87G (Aspartic acid to Glycine) in one isolate (2.2%), *gyrA* D87N (Aspartic acid to Asparagine) and *gyrA* S83Y (Serine to Tyrosine) in two isolates (4.4%), respectively. None of the isolates had a point mutation in the *gyrB, parC,* and *parE* genes (data not shown). Although four isolates exhibited phenotypic resistance to colistin in antimicrobial susceptibility testing, the mobile colistin resistance (*mcr*) gene was not detected in any of them. A single chromosomal mutation, *pmrB* L14F, which is associated with colistin resistance, was identified in one isolate; However, this mutation did not correspond with the observed phenotypic resistance.

Two dominant AMR profiles were identified among the *S.* Enteritidis isolates. The AMP-NAL-STR profile, observed in the majority of isolates (77.8%, 35/45),was associated with the presence of *bla*TEM-1B, *aac(6′)-Iaa, aph(3″)-Ib,* and *sul2,* as well as the *gyrA* D87Y chromosomal mutation. The AMP-FOX-AXO-NAL-STR-TET-STX profile, which conferred resistance to the highest number of antimicrobial classes, was found in 8.9% (4/45) of isolates, and was characterized by *bla*TEM-1B, *bla*CTX-M-14, *fosA3, qnrS1, tet(A), sul2,* and *dfrA17.*

### 3.5 Plasmid replicon profile in *S.* Enteritidis

WGS analysis of 45 *S.* Enteritidis isolates in this study revealed four plasmid incompatibility (Inc) types: IncFIB(S) and IncFII were the most common, detected in 91.1% (41/45), followed by IncX1_4 in 88.9% (32/45), and IncI1-*α* in 11.1% (5/45). Seven distinct Inc. group profiles were identified, as summarized in Table 4 (Supplementary Table S1). The predominant profile -IncFIB(S), IncFII(S), and IncX1- was observed in 71.1% (32/45), followed by IncFIB(S) and IncFII(S) in 8.9% (4/45), and three profiles each accounting for 6.7% (3/45): IncFIB(s), IncFII(S), and IncI1-α; IncFII(S), IncX1; and IncX1 alone. The remaining profiles were each identified in a single isolate, reflecting a diverse plasmid landscape among the isolates.

**Table 4:** Plasmid Inc. group profiles in 45 *S.* Enteritidis isolates in this study.

Inc group profile	Number of isolates (%)
FIB, FII, X1	33 (73.3%)
FIB, FII	4 (8.9%)
FII, FIB, I1-α, X1	3 (6.7%)
X1	3 (6.7%)
FII, FIB, I1-α	1 (2.2%)
I1-α, X1	1 (2.2%)
Total	45 (100%)

### 3.6 wgSNP phylogenetic analysis of *S.* Enteritidis isolates

**Figure 1:**
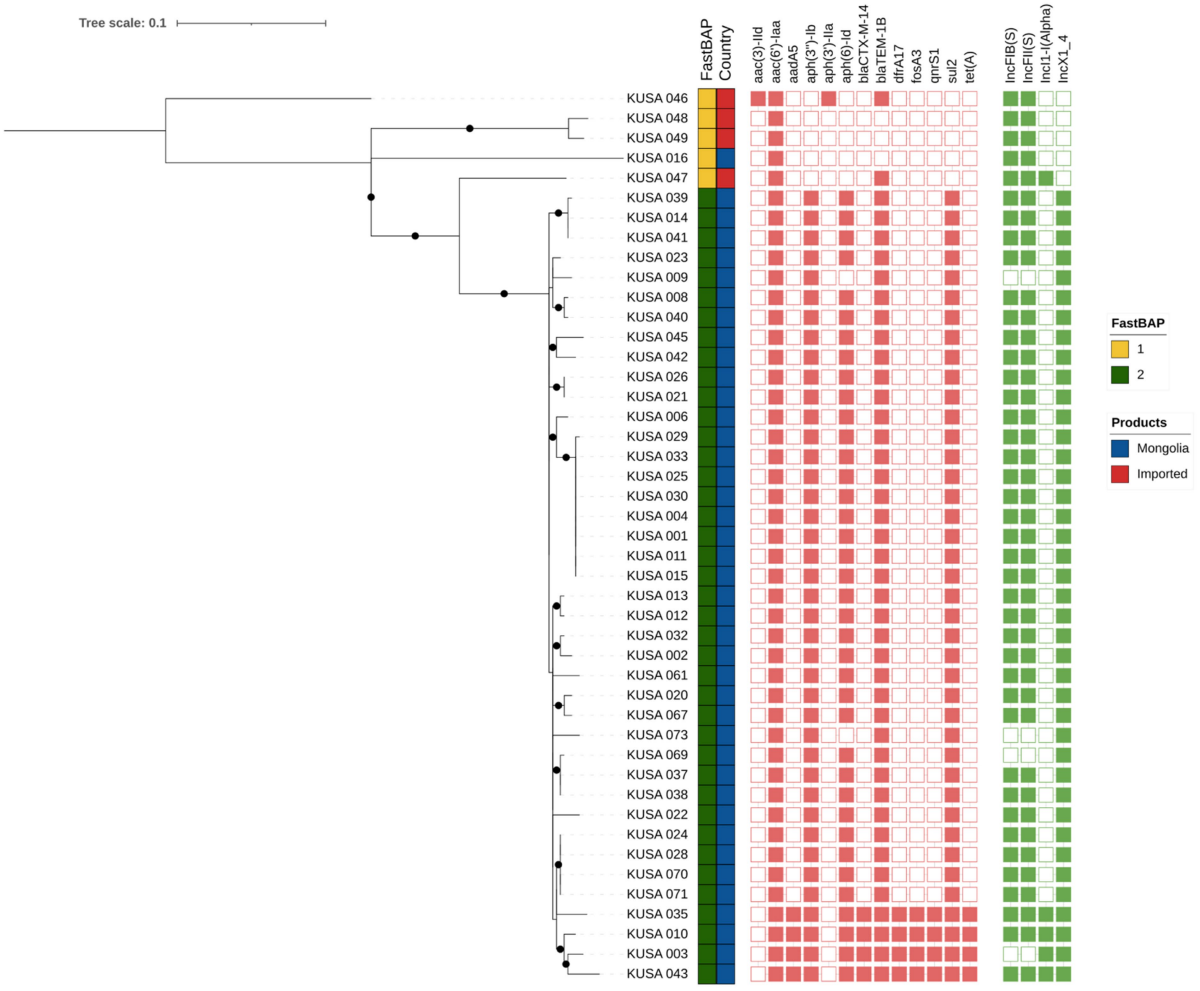
Phylogenetic analysis of 45 *S.* Enteritidis strains isolated in this study. A midpoint-rooted maximum-likelihood (ML) phylogenetic tree was constructed based on whole-genome SNP analysis of 396 SNP sites. The analysis used 1,000 bootstrap replicates and support values greater than 0.9 were indicated by black dots. To determine clustering between *S.* Enteritidis according to genomic similarity, we used FastBAPS. Mongolian domestic chicken isolates were visualized in blue and Chinese imported chicken isolates in red. AMR gene presence was indicated in red, and absence was uncolored. The presence of Plasmid Inc type was indicated in green and absence was uncolored.

A midpoint-rooted maximum-likelihood (ML) phylogenetic tree was constructed based on whole-genome single nucleotide polymorphism (wgSNP) analysis of 396 SNP sites to assess the genetic diversity among 45 *S.* Enteritidis isolates obtained in this study (Figure 1). Based on the SNP alignment and phylogenetic structure, FastBAPS analysis grouped the 45 *S.* Enteritidis isolates into two BAPS clusters: BAPS 1, consisting of Chinese imported isolates, and BAPS 2, comprising Mongolian domestic isolates.

Mongolian domestic *S.* Enteritidis isolates exhibited distinct clustering patterns in the phylogenetic analysis. To further investigate the genetic basis of this separation, AMR gene profiles and plasmid incompatibility (Inc) groups were analyzed, revealing three genetically distinct clusters. Notably, isolates within BAPS 2 (Mongolian domestic group), were further subdivided into a major and minor subgroup based on difference in AMR gene content and Inc. group composition.

The analysis of AMR genes showed that isolates in BAPS 1 harbored fewer AMR genes than those in BAPS 2, with the *aac(6′)-Iaa* gene consistently present in both clusters. Most isolates (35/45) in BAPS 2 group carried the key antimicrobial resistance genes, including *aac(6′)-Iaa, aph(3″)-IIa, aph(6)-Id, bla*TEM-1B, and *sul2*. In contrast, the minor domestic subgroup harbored additional AMR genes, including clinically significant antimicrobial resistance genes like the extended-spectrum beta-lactamase (ESBL) genes *bla*CTX-M-14, as well as plasmid-mediated quinolone resistance (PMQR) genes *qnrS1.*

Plasmid analysis revealed a strong association between Inc. group composition and the distribution of AMR genes. In the BAPS 1, most isolates harbored only IncF plasmid. In contrast, the major Mongolian domestic subgroup primarily exhibited a combination of IncF and IncX1 plasmids, except for three isolates that contained solely IncX1. The minor domestic subgroup also showed a distinct profile characterized by the presence of IncF, IncI1-*α*, and IncX1 plasmids. This variation suggests a potential role of IncI1-α in facilitating the acquisition and dissemination of AMR genes, warranting further investigation.

**Figure 2:**
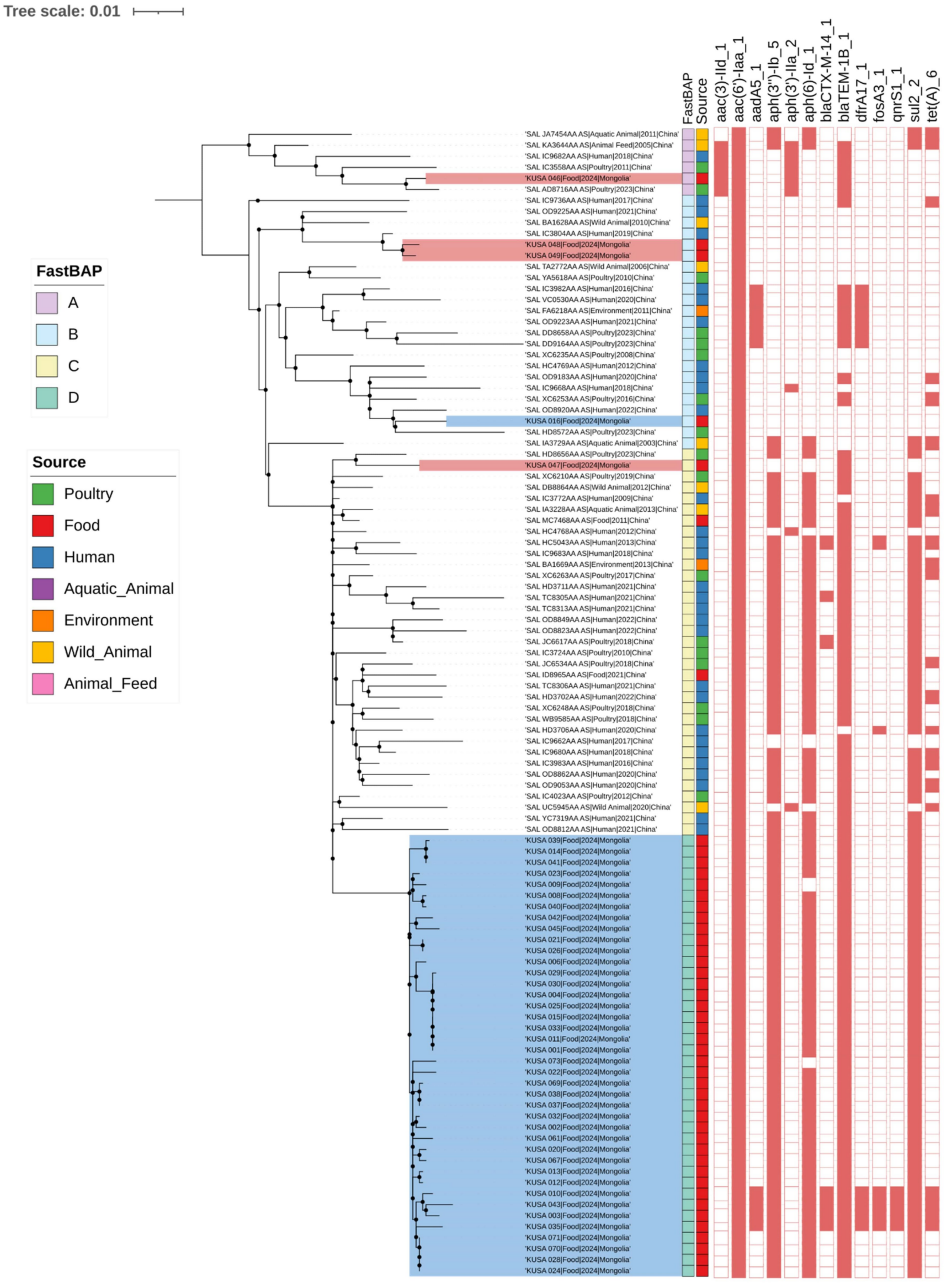
Phylogenetic analysis of 106 *S.* Enteritidis isolate from this study with Chinese *S.* Enteritidis isolates from public database. A midpoint-rooted ML phylogenetic tree was constructed based on whole-genome SNP analysis of 1,502 SNP sites. The analysis used 1,000 bootstrap replicates and support values greater than 0.9 were indicated by black dots. To determine clustering between *S.* Enteritidis according to genomic similarity, we used FastBAPS. Information of source of *S.* Enteritidis was indicated by different colors. The presence of antimicrobial resistance genes is marked with the red square. Antimicrobial resistance gene presence was indicated in red and absence was uncolored.

The distinct clustering profile also highlighted a notable genetic divergence between Mongolian domestic *S.* Enteritidis isolates and those sourced from China, as further supported by a comparative analysis with 61 publicly available Chinese *S.* Enteritidis genomes (Figure 2). A midpoint-rooted ML phylogenetic tree was constructed based on wgSNP analysis of 1,502 SNP sites. FastBAPS grouped the combined dataset into four clusters (BAPS A to D), clearly separating Mongolian isolates (BAPS 2) from Chinese ones (BAPS 1), consistent with their AMR gene profiles and Inc. group compositions.

Notably, all five Chinese imported *S.* Enteritidis isolates clustered closely with isolates from China, indicating high genetic similarity. In BAPS A, one Mongolian domestic isolate (KUSA_046) grouped with four Chinese isolates obtained from animal feed (*n* = 1), aquatic animal (*n* = 1), human (*n* = 1), and poultry (*n* = 2). BAPS B comprised two Chinese imported isolates (KUSA_048 and KUSA_049), which clustered with three Chinese isolates from humans (*n* = 2) and wild animals (*n* = 1), as well as one Mongolian domestic isolate associated with samples from the environment (*n* = 1), human (*n* = 7), wild animal (*n* = 1), and poultry (*n* = 6).

BAPS C included one Chinese imported isolate that clustered with 34 Chinese isolates from various sources. In contrast, BAPS D consisted exclusively of Mongolian domestic isolates and formed a distinct phylogenetic cluster. Despite this separation, BAPS C and BAPS D shared similar AMR gene profiles, including *aac(6′)-Iaa*, *aph(3″)-Ib*, *aph(6)-Id*, *bla*TEM-1B, and *sul2*, which are associated with resistance to aminoglycosides, β-lactams, and sulfonamides.

## 4 Discussion

For the purpose of this study, we collected 114 retail chicken meats -including Mongolian, Chinese and Russian products- from market in Mongolia, with the primary aim of investigating the prevalence of *Salmonella* spp. in poultry meat sold in local retail settings. In addition, further characterization of the isolates was performed through antimicrobial susceptibility testing and WGS.

A previous study in Mongolia employed direct multiplex PCR with specific primers to assess the prevalence of *Salmonella* spp. in poultry meat, reporting a low prevalence of 7.6% (12). In contrast, studies conducted in China have reported a wide range of contamination rates in chicken meat, varying from 10.4 to 57% depending on region and sampling conditions (32–35). The lower prevalence in the prior Mongolian study may be attributed to the use of direct PCR without culture enrichment, which likely reduced the sensitivity of *Salmonella* detection. Pre-enrichment steps using buffered peptone water (BPW) or selenite broth have been shown to significantly improve the sensitivity and specificity of PCR-based detection methods, especially in samples with low bacterial loads, such as food and stool (36–39). Accordingly, standardized culture-based enrichment protocols, such as those recommended by the United States Department of Agriculture Food Safety and Inspection Service (USDA FSIS) Microbiology Laboratory Guidebook (MLG 4.13) (40), are considered essential for accurate *Salmonella* spp. detection. By incorporating standardized enrichment methods, our study achieved a more reliable detection rate, revealing a *Salmonella* spp. prevalence of 49.1%. This isolation rate is notably higher than that reported in previous studies conducted in Mongolia and falls on the higher end of the prevalence range reported in various regions of China. Moreover, *S.* Enteritidis was identified as the predominant serovar, with the majority of isolates classified as MDR. These findings indicate that retail chicken meat sold in Mongolia is frequently contaminated with MDR *Salmonella,* representing a potential threat to food safety and public health.

The wgSNP phylogenetic analysis indicated that 39 *S.* Enteritidis isolates from Mongolian domestic chickens exhibited a monophyletic relationship, characterized by relatively extensive root-to-tip distance and supported by 1,000 bootstrap replicates, in contrast to isolates from imported Chinese poultry (Figure 2). In comparison, a study conducted in the United Kingdom found that *Salmonella* Heidelberg and *S.* Minnesota strains isolated from Brazilian and UK-imported poultry formed a single monophyletic group, distinct from domestic UK strains (41). Unlike the UK, where *Salmonella* from imported poultry was introduced into the local poultry industry, there is no evidence of *S.* Enteritidis transmission from Chinese poultry to Mongolian domestic chickens. This may be explained by Mongolia’s importation of frozen poultry meat rather than live birds (12), which likely reduces the risks of introducing foreign *Salmonella* strains into domestic production. These findings underscore the importance of maintaining and strengthening quarantine and biosecurity measures to prevent the introduction of *Salmonella* and other foodborne pathogens through imported poultry products.

Notably, four isolates within the Mongolian domestic cluster carried IncI1-type plasmids with distinct MDR profiles, including genes conferring resistance to critically important antibiotics such as the ESBL gene *bla*CTX-M-14, and PMQR gene *qnrS1*. The IncI1 plasmid is widely distributed among *Enterobacteriaceae* and has been recognized as a major driver for transferring AMR genes in *Salmonella* spp. (42–44). Globally, IncI1 plasmids carrying *bla*CTX-M-14 have been reported in the UK and Japan (45) and co-carriage of *fosA3* alongside *bla*CTX-M-14 has been documented in China (46), reflecting their widespread role of IncI1 plasmids in AMR dissemination. Although our findings do not confirm that IncI1 plasmids directly carry antimicrobial resistance genes, four *S.* Enteritidis isolates harboring a high number of resistance genes were also found to possess IncI1-type plasmids. This co-occurrence suggests a potential association between IncI1 plasmids and multidrug resistance, which may pose a public health risk if such strains become more prevalent. Therefore, reducing the use of third-generation cephalosporins and quinolones in Mongolia’s poultry industry is crucial to mitigate the potential spread of these MDR strains.

This study has several limitations that should be considered when interpreting the findings. First, the sampling period was relatively short and may not fully capture temporal variation in *Salmonella* prevalence across seasons. Second, the recovery rate of *S.* Enteritidis from Chinese-imported poultry was low, which limited the ability to perform direct genomic comparisons with domestic strains. To partially address this, we supplemented our analysis with publicly available *S.* Enteritidis genomes from EnteroBase, enabling broader phylogenetic contextualization and comparison.

Given the high prevalence of MDR *S.* Enteritidis among Mongolian isolates, including resistance to clinically important antibiotics such as ESBLs and PMQR, and the increasing consumption of poultry meat in Mongolia, the implementation of effective antibiotic stewardship in domestic poultry production is urgently needed to mitigate potential public health risks. To support these efforts, future studies should aim to conduct longitudinal and geographically broader surveillance to better understand temporal and spatial trends in *Salmonella* contamination.

In conclusion, this study represents the first prevalence-based investigation of *S.* Enteritidis isolates from poultry in Mongolia, providing valuable insights into their genetic diversity, antimicrobial resistance profiles, and phylogenetic relationships. These finding establish critical baseline data to inform future monitoring and control strategies to combat the spread of MDR *S.* Enteritidis in the Mongolian poultry industry.

## Data Availability

The whole-genome sequencing data generated in this study has been deposited in the NCBI BioProject database under accession number PRJNA1236736.

## Footnotes

^1^ http://github.com/tseemann/snippy

^2^https://itol.embl.de; accessed on 21, February 2025

^3^ https://enterobase.warwick.ac.uk

^4^ github.com/tseemann/abricate

^5^ github.com/maxlcummins/abricateR

## References

[1] Teklemariam AD Al-Hindi RR Albiheyri RS Alharbi MG Alghamdi MA Filimban AAR. Human salmonellosis: a continuous global threat in the farm-to-fork food safety continuum. Food Secur. (2023) 12:1756. doi: 10.3390/foods12091756, PMID: 37174295 PMC10178548

[2] Sher AA Mustafa BE Grady SC Gardiner JC Saeed AM. Outbreaks of foodborne *Salmonella enteritidis* in the United States between 1990 and 2015: an analysis of epidemiological and spatial-temporal trends. Int J Infect Dis. (2021) 105:54–61. doi: 10.1016/j.ijid.2021.02.022 33578006

[3] Peng S Xiong H Lu J Luo F Liu C Zhou H. Epidemiological and whole genome sequencing analysis of restaurant *Salmonella Enteritidis* outbreak associated with an infected food handler in Jiangxi Province, China, 2023. Foodborne Pathog Dis. (2024) 21:316–22. doi: 10.1089/fpd.2023.0123, PMID: 38354216

[4] About National Enteric Disease Surveillance | National Surveillance of Bacterial Foodborne Illnesses (Enteric Diseases) | CDC. Available online at: https://www.cdc.gov/national-enteric-surveillance/about/index.html?CDC_AAref_Val=https://www.cdc.gov/nationalsurveillance/pdfs/2016-Salmonella-report-508.pdf (Accessed March 10, 2025).

[5] Munkhdelger B. The meat processing industry in Mongolia. Int J Scientific Res Public. (2020) 10:563–568. doi: 10.29322/ijsrp.10.03.2020.p9963

[6] Wang W Zhao L Hu Y Dottorini T Fanning S Fanning S. Epidemiological study on prevalence, serovar diversity, multidrug resistance, and CTX-M-type extended-spectrum β-lactamases of salmonella spp. from patients with diarrhea, food of animal origin, and pets in several provinces of China. Antimicrob Agents Chemother. (2020) 64:e00092-20. doi: 10.1128/AAC.00092-20 32312775 PMC7318004

[7] Wang W Cui J Liu F Hu Y Li F Zhou Z. Genomic characterization of Salmonella isolated from retail chicken and humans with diarrhea in Qingdao, China. Front Microbiol. (2023) 14:1295769. doi: 10.3389/fmicb.2023.1295769, PMID: 38164401 PMC10757937

[8] Wang Z Jiang Y Xu H Jiao X Wang J Li Q. Poultry production as the main reservoir of ciprofloxacin- and tigecycline-resistant extended-spectrum β-lactamase (ESBL)producing *Salmonella enterica* serovar Kentucky ST198.2-2 causing human infections in China. Appl Environ Microbiol. (2023) 89:e0094423. doi: 10.1128/aem.00944-23, PMID: 37610223 PMC10537671

[9] Cooper AL Low AJ Koziol AG Thomas MC Leclair D Tamber S. Systematic evaluation of whole genome sequence-based predictions of Salmonella serotype and antimicrobial resistance. Front Microbiol. (2020) 11:549. doi: 10.3389/fmicb.2020.00549, PMID: 32318038 PMC7147080

[10] Deng Y Jiang M Kwan PSL Yang C Chen Q Lin Y. Integrated whole-genome sequencing infrastructure for outbreak detection and source tracing of *Salmonella enterica* serotype Enteritidis. Foodborne Pathog Dis. (2021) 18:582–9. doi: 10.1089/fpd.2020.2856, PMID: 33450161

[11] Hu L Cao G Brown EW Allard MW Ma LM Zhang G. Whole genome sequencing and protein structure analyses of target genes for the detection of Salmonella. Sci Rep. (2021) 11:20887. doi: 10.1038/s41598-021-00224-7, PMID: 34686701 PMC8536731

[12] Zhanabayeva DK Paritova AY Murzakaeva GK Zhanabayev AA Kereev A Asauova ZS. Pcr diagnosis for the identification of the virulent gene of salmonella in poultry meat. Online J Biol Sci. (2021) 21:235–44. doi: 10.3844/ojbsci.2021.235.244

[13] Chen S Zhou Y Chen Y Gu J. Fastp: an ultra-fast all-in-one FASTQ preprocessor. Bioinformatics. (2018) 34:i884–90. doi: 10.1093/bioinformatics/bty560, PMID: 30423086 PMC6129281

[14] Bolger AM Lohse M Usadel B. Trimmomatic: a flexible trimmer for Illumina sequence data. Bioinformatics. (2014) 30:2114–20. doi: 10.1093/bioinformatics/btu170, PMID: 24695404 PMC4103590

[15] Bankevich A Nurk S Antipov D Gurevich AA Dvorkin M Kulikov AS. SPAdes: a new genome assembly algorithm and its applications to single-cell sequencing. J Comput Biol. (2012) 19:455–77. doi: 10.1089/cmb.2012.0021, PMID: 22506599 PMC3342519

[16] Zhang S den Bakker HC Li S Chen J Dinsmore BA Lane C. SeqSero2: rapid and improved salmonella serotype determination using whole-genome sequencing data. Appl Environ Microbiol. (2019) 85:e01746-19. doi: 10.1128/AEM.01746-19, PMID: 31540993 PMC6856333

[17] NARMS. National Antimicrobial Resistance Monitoring System (NARMS): 2014 human isolates final report. (2016) Available online at: https://stacks.cdc.gov/view/cdc/41823 (Accessed March 10, 2025)

[18] Limbago B. CLSI. Performance standards for antimicrobial susceptibility testing, vol. 23. 29th ed. CLSI supplement M100. Wayne, PA: Clinical and Laboratory Standards Institute (2019).

[19] Magiorakos AP Srinivasan A Carey RB Carmeli Y Falagas ME Giske CG. Multidrug-resistant, extensively drug-resistant and pandrug-resistant bacteria: an international expert proposal for interim standard definitions for acquired resistance. Clin Microbiol Infect. European Society of Clinical Microbiology and Infectious Diseases (ESCMID), published by Elsevier on behalf of ESCMID. (2012) 18:268–81. doi: 10.1111/j.1469-0691.2011.03570.x, PMID: 21793988

[20] Seemann T. Snippy, Rapid haploid variant calling and core genome alignment. *GitHub repository* (2015)

[21] Wishart DS Han S Saha S Oler E Peters H Grant JR. PHASTEST: faster than PHASTER, better than PHAST. Nucleic Acids Res. (2023) 51:W443–50. doi: 10.1093/nar/gkad382, PMID: 37194694 PMC10320120

[22] Marçais G Delcher AL Phillippy AM Coston R Salzberg SL Zimin A. MUMmer4: A fast and versatile genome alignment system. PLoS Comput Biol. (2018) 14:e1005944. doi: 10.1371/journal.pcbi.1005944, PMID: 29373581 PMC5802927

[23] Croucher NJ Page AJ Connor TR Delaney AJ Keane JA Bentley SD. Rapid phylogenetic analysis of large samples of recombinant bacterial whole genome sequences using Gubbins. Nucleic Acids Res. (2015) 43:e15. doi: 10.1093/nar/gku1196, PMID: 25414349 PMC4330336

[24] Kalyaanamoorthy S Minh BQ Wong TKF Von Haeseler A Jermiin LS. ModelFinder: fast model selection for accurate phylogenetic estimates. Nat Methods. (2017) 14:587–9. doi: 10.1038/nmeth.4285, PMID: 28481363 PMC5453245

[25] Nguyen LT Schmidt HA Von Haeseler A Minh BQ. IQ-TREE: a fast and effective stochastic algorithm for estimating maximum-likelihood phylogenies. Mol Biol Evol. (2015) 32:268–74. doi: 10.1093/molbev/msu300, PMID: 25371430 PMC4271533

[26] Achtman M Zhou Z Alikhan NF Tyne W Parkhill J Cormican M. Genomic diversity of *Salmonella enterica* -the UoWUCC 10K genomes project. Wellcome Open Res. (2021) 5:223. doi: 10.12688/wellcomeopenres.16291.2, PMID: 33614977 PMC7869069

[27] Katz L Griswold T Morrison S Caravas J Zhang S Bakker H. Mashtree: a rapid comparison of whole genome sequence files. J Open Source Softw. (2019) 4:1762. doi: 10.21105/joss.01762, PMID: 35978566 PMC9380445

[28] Menardo F Loiseau C Brites D Coscolla M Gygli SM Rutaihwa LK. Treemmer: a tool to reduce large phylogenetic datasets with minimal loss of diversity. BMC Bioinformatics. (2018) 19:164. doi: 10.1186/s12859-018-2164-8, PMID: 29716518 PMC5930393

[29] Tonkin-Hill G Lees JA Bentley SD Frost SDW Corander J. Fast hierarchical Bayesian analysis of population structure. Nucleic Acids Res. (2019) 47:5539–49. doi: 10.1093/nar/gkz361, PMID: 31076776 PMC6582336

[30] Bortolaia V Kaas RS Ruppe E Roberts MC Schwarz S Cattoir V. ResFinder 4.0 for predictions of phenotypes from genotypes. J Antimicrob Chemother. (2020) 75:3491–500. doi: 10.1093/jac/dkaa345, PMID: 32780112 PMC7662176

[31] Carattoli A Zankari E Garciá-Fernández A Larsen MV Lund O Villa L. PlasmidFinder and pMLST: in silico detection and typing of plasmid. Antimicrob Agents Chemother. (2014) 58:3895–903. doi: 10.1128/AAC.02412-14, PMID: 24777092 PMC4068535

[32] Sun T Liu Y Qin X Aspridou Z Zheng J Wang X. The prevalence and epidemiology of salmonella in retail raw poultry meat in China: a systematic review and meta-analysis. Food Secur. (2021) 10:2757. doi: 10.3390/foods10112757, PMID: 34829037 PMC8622452

[33] Liu C Yao K Ren D Xiao Y. Prevalence and characterization of Salmonella from meat in slaughterhouses in Hangzhou, China. Int J Food Microbiol. (2022) 371:109649. doi: 10.1016/j.ijfoodmicro.2022.109649, PMID: 35468523

[34] Yang X Huang J Zhang Y Liu S Chen L Xiao C. Prevalence, abundance, serovars and antimicrobial resistance of Salmonella isolated from retail raw poultry meat in China. Sci Total Environ. (2020) 713:136385. doi: 10.1016/j.scitotenv.2019.136385, PMID: 31955074

[35] Gu D Wang Z Tian Y Kang X Meng C Chen X. Prevalence of Salmonella isolates and their distribution based on whole-genome sequence in a chicken slaughterhouse in Jiangsu, China. Front Vet Sci. (2020) 7:29. doi: 10.3389/fvets.2020.00029, PMID: 32154275 PMC7046563

[36] Vinayaka AC Ngo TA Kant K Engelsmann P Dave VP Shahbazi MA. Rapid detection of *Salmonella enterica* in food samples by a novel approach with combination of sample concentration and direct PCR. Biosens Bioelectron. (2019) 129:224–30. doi: 10.1016/j.bios.2018.09.078, PMID: 30318404

[37] Chirambo AC Nyirenda TS Jambo N Msefula C Kamng’ona A Molina S. Performance of molecular methods for the detection of Salmonella in human stool specimens. Wellcome Open Res. (2021) 5:237. doi: 10.12688/wellcomeopenres.16305.2, PMID: 34017923 PMC8108707

[38] Parker AM Mohler VL Gunn AA House JK. Development of a qPCR for the detection and quantification of Salmonella spp. in sheep feces and tissues. J Vet Diagn Invest. (2020) 32:835–43. doi: 10.1177/1040638720952359, PMID: 32856555 PMC7649550

[39] Ríos-Castillo AG Ripolles-Avila C Rodríguez-Jerez JJ. Detection of Salmonella typhimurium and *Listeria monocytogenes* biofilm cells exposed to different drying and pre-enrichment times using conventional and rapid methods. Int J Food Microbiol. (2020) 324:108611. doi: 10.1016/j.ijfoodmicro.2020.108611, PMID: 32229312

[40] Rose BE. Isolation and identification of Salmonella from meat, poultry, pasteurized egg, and catfish products and carcass and environmental sponges. Washington, DC, USA: USDA Microbiology Laboratory Guidebook (2014).

[41] Alikhan NF Moreno LZ Castellanos LR Chattaway MA McLauchlin J Lodge M. Dynamics of *Salmonella enterica* and antimicrobial resistance in the Brazilian poultry industry and global impacts on public health. PLoS Genet. (2022) 18:e1010174. doi: 10.1371/journal.pgen.1010174, PMID: 35653335 PMC9162342

[42] Oladeinde A Abdo Z Press MO Cook K Cox NA Zwirzitz B. Horizontal gene transfer is the Main driver of antimicrobial resistance in broiler chicks infected with *Salmonella enterica* Serovar Heidelberg. mSystems. (2021) 6:e00729-21. doi: 10.1128/msystems.00729-21 34427525 PMC8409728

[43] Carattoli A Villa L Fortini D García-Fernández A. Contemporary IncI1 plasmids involved in the transmission and spread of antimicrobial resistance in Enterobacteriaceae. Plasmid. (2021) 118:102392. doi: 10.1016/j.plasmid.2018.12.001, PMID: 30529488

[44] Foley SL Kaldhone PR Ricke SC Han J. Incompatibility group I1 (IncI1) plasmids: their genetics, biology, and public health relevance. Microbiol Mol Biol Rev. (2021) 85:e00031-20. doi: 10.1128/mmbr.00031-20, PMID: 33910982 PMC8139525

[45] Kameyama M Chuma T Yokoi T Yabata J Tominaga K Miyasako D. Emergence of *Salmonella enterica* serovar infantis harboring IncI1 plasmid with blaCTX-M-14 in a broiler farm in Japan. J Vet Med Sci. (2012) 74:1213–6. doi: 10.1292/jvms.11-0488 22673563

[46] Wang XM Dong Z Schwarz S Zhu Y Hua X Zhang Y. Plasmids of diverse Inc groups disseminate the fosfomycin resistance gene fosA3 among *Escherichia coli* isolates from pigs, chickens, and dairy cows in Northeast China. Antimicrob Agents Chemother. (2017) 61:e00859-17. doi: 10.1128/AAC.00859-17, PMID: 28674050 PMC5571358

[47] Eng SK Pusparajah P Ab Mutalib NS Ser HL Chan KG Lee LH. Salmonella: a review on pathogenesis, epidemiology and antibiotic resistance. Front Life Sci. (2015) 8:284–93. doi: 10.1080/21553769.2015.1051243, PMID: 40101104

